# Use of Online Tools for Mental Health Among Racially and Ethnically Diverse College Students: Mixed Methods Study

**DOI:** 10.2196/60628

**Published:** 2025-06-13

**Authors:** Sarah Z Hamza, Yesenia Aguilar Silvan, Lauren C Ng

**Affiliations:** 1 University of California, Los Angeles Los Angeles, CA United States

**Keywords:** mental health, online information, online tool, college students, help-seeking behavior, survey, qualitative, literacy, experience, attitude, perspective, self-efficacy, diverse

## Abstract

**Background:**

Anxiety and depression symptoms have been rising among college students, with many increasingly meeting the criteria for 1 or more mental health problems. Due to a rise in internet access and lockdown restrictions associated with the COVID-19 pandemic, online mediums, such as teletherapy, repositories for mental health information, discussion forums, self-help programs, and online screening tools, have become more popular and used by college students to support their mental health. However, there is limited information about individual-level factors that lead college students to use these online tools to support their mental health.

**Objective:**

This mixed methods study aimed to examine the associations between demographics, symptom severity, mental health literacy, stigma, attitudes, and self-efficacy and the use of online tools to seek psychological information and services among racially and ethnically diverse college students. This study also aimed to qualitatively characterize types of online tools used, reasons for using tools or lack thereof, and perceived helpfulness of tools.

**Methods:**

Undergraduate students (N=123) completed validated measures and provided open-ended descriptions of the types of online tools they used to seek psychological information and services and their reasons for using those tools. Logistic regression analyses were used to test associations of online tool use to seek mental health information and hypothesized predictors. Descriptive statistics were conducted to examine online tool types, reasons for using online tools, and helpfulness explanations.

**Results:**

In total, 49.6% (61/123) of the participants used online tools (eg, search engines) to seek mental health information, while 30.1% (37/123) used online tools (eg, medical websites) to seek mental health services. Mental health literacy (*P*=.002; odds ratio 1.14, 95% CI 1.05-1.24) was associated with greater use of online tools to seek mental health information. None of the hypothesized variables predicted online tool use to seek mental health services. In total, 82% (50/61) of participants who sought information found online tools somewhat helpful, while 49% (18/37) of participants who sought services found online tools very helpful. Of the students who did not use online tools to seek information, 19% (12/62) reported it was because they did not know which online tools to use and 31% (19/62) stated they would be encouraged to use online tools if it was recommended by professionals, therapists, family, or friends. Of the students who did not use online tools to seek services, 33% (28/86) reported it was because they did not think mental health help was necessary.

**Conclusions:**

These findings highlight the use of online tools to provide mental health information and connect to professional services, suggesting that online tools are widely used to access mental health support.

## Introduction

### Mental Health Prevalence and Service Use

Due to an increase in the prevalence of mental disorders among college students over the past decade, exploring and addressing mental health concerns has become highly necessary. In 2024, approximately 70% of college students reported mild to severe depression or anxiety, which is a substantial increase in depression and anxiety symptoms since 2013 [1,2]. College students have reported that mental health problems affected their academic success, with depression symptoms predicting their grade point average and likelihood of dropping out, further highlighting the need to increase access to and use of effective mental health treatments among this population [3,4]. While past-treatment use for mental health problems has increased among college students, young people (aged 18-25 years) have the lowest rates of mental care use among the adult population [1,2,5]. Furthermore, racial or ethnic minority (eg, Black, multiracial, and Arab American) college students have lower rates of mental health care use, including psychological and pharmacological treatment, compared to non-Hispanic White college students, resulting in a need to examine novel tools that can increase mental health service use rates and potentially offset barriers to care for racial or ethnic minority college students [2].

### Digital Help-Seeking Behaviors

One way to increase mental health service use rates is to improve help-seeking behaviors, which refers to the act of seeking out and using formal sources (eg, clinical psychologist), semiformal sources (eg, teachers and clergy), informal sources (eg, friends and family), and self-help (eg, unguided website use) sources to address mental health problems [6]. Due to a rise in internet access and lockdown restrictions associated with the COVID-19 pandemic worldwide, online mediums such as virtual counseling and therapy services, repositories for mental health information and resources, discussion forums, self-help programs, and online screening tools have become more popular among college students to support their mental health [7,8]. Global initiatives (eg, World Mental Health International College Student initiative) have prioritized focusing on research that leverages digital mental health interventions to support young peoples’ mental health, further demonstrating the potential of digital technologies and online resources to increase service use among college students and potentially address inequities in care access [9,10]. Despite the increased use and research prioritization of online resources to support college students’ mental health, there is limited information about individual-level factors that lead college students to use online tools for mental health support. In addition, there is little research examining why college students might opt out of using online tools for mental health support.

### Predictors of Digital Tool Use

#### Past Therapy and Symptom Severity

Research shows that young adults who had previously used formal counseling services started to use those services digitally during the COVID-19 pandemic to support their mental health as they transitioned to online spaces [8]. Positive past experiences with mental health services were associated with greater engagement in digital mental health interventions [11]. Furthermore, a systematic review found that globally, students who had received a mental health diagnosis in the past and wanted to monitor their mental status were more likely to use online mediums compared to those without a diagnosis [7]. Even among students without a mental health diagnosis, being curious about their symptoms or having severe symptoms increased students’ likelihood of using online tools, such as educational materials, self-help interventions, screening tools, and online therapies delivered by professionals, for their mental health [7,12,13].

#### Stigma and Attitudes

Worldwide, stigma is a well-documented barrier to traditional mental health help seeking, often deterring individuals from pursuing care due to concerns about disclosure and social judgment [14]. However, the anonymity, immediacy, and confidentiality of online platforms may reduce concerns about disclosure and social stigma, possibly making them a more accessible option for help seeking [14]. Unlike traditional help seeking, research suggests that stigma does not significantly impact the use of online self-help resources [15]. Similarly, negative attitudes toward mental health or external influences from others do not appear to deter online help-seeking intentions among young people [16,17]. Nevertheless, more research is needed to understand why these factors may not have a strong influence on online help-seeking behaviors.

#### Mental Health Literacy and Self-Efficacy

Despite the accessibility and anonymity of online tools, barriers such as low mental health literacy continue to limit their use among college students [7,14]. Students with limited mental health literacy may lack the knowledge needed to recognize their symptoms and identify available services. Without this essential knowledge, students may struggle to initiate the online search process, including identifying relevant keywords or finding evidence-based mental health resources and tools online. Another barrier to using online tools may be due to students’ self-efficacy [18]. While studies worldwide have explored self-efficacy as a predictor of traditional help-seeking behaviors within this population, research examining the role of self-efficacy in online tool use remains limited [19]. Further investigation is needed to better understand this relationship.

#### Race or Ethnicity, Gender, Age, and Non–US Born Status

It is possible that there are disparities in the use of online tools based on college students’ demographics. One study found that racial and ethnic minority students expressed more interest in teletherapy and online self-help guided mental health support when they were free compared to non-Hispanic White students, but their interest declined when a cost was involved [16]. However, another study found that racial and ethnic minority students were just as likely as their non-Hispanic White counterparts to enroll in and initiate teletherapy [20]. There are also mixed results on the association between gender and use of online tools for mental health, with some studies reporting no gender differences and others reporting that women are more likely to use online tools than men [7,20,21]. In terms of age, younger adults are more likely to use smartphone apps for their mental health or well-being compared to older adults [22]. Finally, while non–US born immigrants are more reluctant to partake in traditional mental health help-seeking behaviors due to stigma and language barriers, research exploring the relationship between non–US born status and digital help-seeking behavior is limited [23]. For immigrants and refugees, digital tools may still impose barriers to help seeking due to limited language services and the cultural appropriateness of content [24]. These mixed and limited findings highlight the need to explore disparities in the use of digital mental health tools among students from diverse racial and ethnic, gender, and non–US backgrounds.

### Objectives of the Study

The relationship between individual-level factors such as symptom severity, stigma, mental health literacy, and online tool use among university students still needs to be further explored to better understand how digital tools can be leveraged to promote mental health equity among college students [21,24]. Consequently, we first examined the association between race and ethnicity, non–US born status, gender, symptom severity, mental health literacy, stigma, self-efficacy, and attitudes toward mental health treatment and the use of online tools for mental health. Then, we identified themes related to factors influencing online tool use and perceptions of the helpfulness of online tools by exploring the types of online tools used, reasons for using or not using online tools, helpfulness of online tools, and motivators for using online tools in the future.

### Hypotheses of the Study

This study had four primary hypotheses: (1) we hypothesized that racial and ethnic minority and non–US born students would be more likely to use online tools to seek help due to digital tools addressing stigma-related barriers hindering traditional help seeking among these students [14,25,26]; (2) we also hypothesized that younger students and women would be more likely to use online tools due to existing research showcasing that they are more likely to use online mental health tools, such as smartphone apps, discussion forums, and internet searches [21,22]; (3) we hypothesized that more severe symptoms of depression, anxiety, and trauma would each be independently associated with a greater likelihood of using online tools to seek help as well because symptom severity predicts students’ use of different online mental health resources [7,12]; (4) finally, we hypothesized that lower mental health stigma, higher mental health literacy, greater self-efficacy, and more positive attitudes toward help seeking would each be independently associated with a greater likelihood of using online tools for mental health support, as these factors predict traditional help-seeking behaviors [14,18,19,25].

## Methods

### Study Design and Sample

This study is a cross-sectional mixed methods analysis derived from a larger study examining the use of direct-to-consumer strategies to increase initial engagement in mental health services. Participants (N=123) were recruited through the University of California, Los Angeles (UCLA) Psychology Subject Pool, a university system that allows researchers to recruit participants for research studies, from May 2021 to December 2021. Data collection occurred during the COVID-19 pandemic, during which there were lockdown restrictions in the United States, such as school and nonessential business closures, large gathering bans, and stay-at-home orders [27]. The study was designed to be completed entirely online. The UCLA Psychology Subject Pool system was used to post study advertisements with the inclusion criteria and allow participants to sign up for the study on a secure online platform. The inclusion criteria included undergraduate students at UCLA who were interested in seeking mental health services, were aged at least 18 years, and had corrected to normal vision. The participants represent UCLA’s student body, which is racially and ethnically diverse, with approximately 75% of the undergraduate population reporting a racial or ethnic minority identity [28]. In addition, around 15% of the student population at UCLA has indicated the need to use mental health services in the past [29].

### Ethics Approval

All study procedures were approved by the UCLA Institutional Review Board (IRB#20-002082). After signing up for the study on the UCLA Psychology Subject Pool system, participants met with an undergraduate-level research assistant through Health Insurance Portability and Accountability Act–compliant Zoom (Zoom Communications, Inc) video call to discuss consent procedures. Only after consenting to participate in the study were participants allowed to complete a series of surveys. Students received 1 course credit for their participation in completing the surveys. Collected data were deidentified and securely stored. Only authorized personnel had access to the data.

### Measures

#### Demographic Form

Participants completed a 14-item self-reported demographic form. Demographic variables included age, gender, assigned sex at birth, race, ethnicity, place of birth, total household income during childhood, and past experiences with medication and the use of therapy or counseling for mental health treatment. Race or ethnicity were categorized as racial and ethnic minorities and White for our sample. Middle Eastern was categorized as a racial or ethnic minority, alongside Asian, Latinx, Black or African American, and multiracial. This categorization aligns with research findings indicating that the designation of “White” does not correspond to Middle Easterners’ lived experiences or others’ perceptions of them [30]. One participant did not report their race or ethnicity, resulting in 1 missing value for the race or ethnicity demographic variable.

#### Patient Health Questionnaire-9

The Patient Health Questionnaire-9 comprises 9 questions designed to detect the 9 diagnostic criteria for major depressive disorder according to the *Diagnostic and Statistical Manual of Mental Disorders, 4th Edition* [31]. Responses to these questions were measured on a 4-point scale from 0 to 3, where 0 indicated “never” and 3 represented “nearly every day.” To comply with the institutional review board requirements, item 9, which assesses suicidality, was removed. The total score was calculated by combining reported values from all 8 questions. In this sample, reliability was fairly high (α=.88) [32]. There were no missing data for this variable.

#### General Anxiety Disorder-7

The General Anxiety Disorder-7 questionnaire consists of 7 questions used to measure each 1 of the 7 core symptoms of generalized anxiety disorder [33]. Responses to these questions were measured using a 4-point scale from 0 to 3, where 0 indicated “never” and 3 represented “nearly every day.” The total score was calculated by combining reported values from all 7 questions. In this sample, reliability was fairly high (α=.90) [32]. There were no missing data for this variable.

#### PTSD Checklist for DSM-5

The PTSD Checklist for the DSM-5 comprises 20 questions, each designed to measure 1 of the 20 corresponding posttraumatic stress disorder (PTSD) symptom criteria outlined in the DSM-5 [34]. Responses for these questions were measured using a 4-point scale from 0 to 3, with 0 indicating “not at all” and 4 referring to “extremely” in terms of how bothered the individual feels by the given PTSD symptom over the past month. The total score was calculated, combining reported values from all 20 questions. In this sample, reliability was deemed fairly high (α=.95) [32]. There were no missing data for this variable.

#### Mental Health Literacy Scale

The mental health literacy scale consists of 31 questions used to measure the mental health literacy of individuals [35]. The first 15 items were measured using a 4-point scale from 0 to 3, with 0 indicating “very unlikely” or “very unhelpful” and 3 referring to “very likely” or “very helpful.” The rest of the questions were scored on a 5-point scale from 0 to 4, with 0 indicating “strongly disagree” and 4 indicating “strongly agree.” The total score was calculated by combining reported values from all 31 questions. Our sample indicated fairly high reliability (α=.82) [32]. This scale was incorporated into the original study at a later stage, resulting in missing data for 29% (36/123) of the participants who did not receive this measure.

#### Internalized Stigma of Mental Illness Scale-10

The Internalized Stigma of Mental Illness-10 scale comprises 10 questions designed to measure the extent of internalized stigma among individuals with psychiatric disorders; responses are rated on a 4-point Likert scale, ranging from 1 (strongly disagree) to 4 (strongly agree) [36]. The total score was calculated by combining reported values for the 10 questions. Our sample had high reliability (α=.74) [32]. There were no missing data for this variable.

#### Attitudes Toward Mental Health Treatment Scale

The Attitudes Toward Mental Health Treatment Scale consists of 20 questions and is used to assess internalized stigma and attitudes and behaviors related to seeking mental health treatment [37]. Responses to these questions were measured using a 4-point Likert scale, where participants ranked their level of agreement with statements about mental health treatment, with 1 indicating “strongly disagree” and 4 representing “strongly agree”. The total score was calculated by combining reported values from all 20 questions. Our sample also indicated fairly high reliability (α=.78) [32]. There were no missing data for this variable.

#### Self-Efficacy in Seeking Mental Health Care Scale

The Self-Efficacy in Seeking Mental Health Care Scale consists of 9 questions used to measure self-efficacy for mental health care by assessing 2 subscales: confidence in knowledge and confidence in coping [38]. Responses to these questions were measured using a Likert response scale ranging from 1 to 10, where 1 represents no confidence and 10 indicates complete confidence in the individual’s ability to conduct a given behavior. For this study, only the total score was calculated by combining reported values from all 9 questions. Finally, our sample had fairly high reliability (α=.89) [32]. There were no missing data for this variable.

#### Online Tool Use

Questions from the California Health Interview Survey were used to assess online tool use [39]. Participants were asked 2 dichotomous (ie, yes or no) questions: “Have you used an online tool (eg, mobile apps, texting services, webpages, or forums) to learn about problems with your mental health, emotions, nerves, or your use of alcohol/drugs?” and “Have you used online tools (eg, mobile apps, texting services, or webpages) to find, be referred to, contact, or connect with a mental health professional/service?” In addition, participants were asked to rate the helpfulness of the online tools, with response options being very helpful, somewhat helpful, or not at all helpful. There were no missing data for these variables.

To obtain more detailed information about online tool use, participants who responded “yes” were asked to provide open-ended descriptions of the type of online tools used, reasons for their use, and why they were helpful. Those who responded “no” were asked to share open-ended descriptions of reasons for not using online tools and the motivators that would encourage them to use such tools for learning about mental health problems or connecting with mental health services. Open-ended responses varied in length, with some participants providing brief 3- to 4-word answers, while others offered more detailed responses consisting of a few sentences.

### Statistical Analyses

#### Mixed Methods Design

A Qual+QUAN mixed methods design was used to analyze the data [40]. This design incorporated simultaneous collection and analysis of both quantitative and qualitative data for confirmation and hypothesis testing. Quantitative data analysis was the primary method, examining a priori constructs that could impact online tool use. The qualitative data expanded upon the quantitative findings, such that it provided a deeper understanding of the relationship between a priori constructs and online tool use. Specifically, the qualitative analysis aimed to identify themes related to factors influencing online tool use and perceptions of their helpfulness by exploring the types of online tools participants used and the reasons behind their use.

#### Quantitative Analysis

To analyze sample characteristics, descriptive analysis was conducted using jamovi (version 2.3; the jamovi project [41]). In addition, associations between student-level factors and the use of online tools for mental health were examined using multiple logistic regressions. These regressions assessed the relationship between demographics, symptom severity, mental health literacy, stigma, attitudes toward mental health treatment, self-efficacy, and engagement with online tools for seeking mental health information and professional services. Finally, due to previous research showcasing past therapy use as one of the few consistent predictors of online tool use for mental health, we controlled for past therapy use for the regression analyses [7,8,11].

#### Qualitative Analysis

Qualitative data underwent an inductive (“bottom-up”) approach, creating codes based directly on the participants’ open-ended text responses without applying a preexisting theoretical framework or a priori codes [42]. The first author familiarized themselves with the data, taking notes and highlighting important points. Using 20% of the data, initial codes were generated based on emerging concepts, such as reasons for using or not using online tools. These codes were categorized based on how responses were related (eg, “gave me helpful techniques to calm myself down” and “it taught me how to breathe in stressful situations” were combined into the code “coping mechanisms”) and refined iteratively by the first and second authors in weekly coding meetings throughout March 2023. Definitions were developed for each code and category, which were then grouped into themes to create a final codebook. The final codebook consisted of a label, definitions, and exemplar statements derived from the data. The first author then coded the open-ended responses based on the final codebook. Coding decisions were reviewed during consensus meetings held with the second author, which is an approach commonly used to address coding biases and ensure coding reliability [43]. Open-ended responses that were not consistent with the codebook definitions were also discussed during consensus meetings. When responses were too vague (eg, “online resource” and “I don’t know”), it was decided to categorize those statements as “vague” rather than forcing them into previously established categories in the final codebook. After coding all the responses, the codes and categories were interpreted at the theme level. Thematic analysis allowed us to identify overarching patterns within the data to better understand factors influencing online tool use and perceptions of the helpfulness of online tools.

## Results

### Sample Characteristics

A total of 123 participants completed the survey and were aged on average 21 (SD 3.39) years. The majority self-identified as women (104/123, 84.6%) and belonged to racial or ethnic minority groups (98/123, 79.7%). Racial or ethnic minority participants were primarily Asian (47/123, 38.2%) followed by Middle Eastern (19/123, 15.4%), Latinx (16/123, 13%), multiracial (11/123, 9%), and Black or African American (5/123, 4.1%). Furthermore, a substantial proportion of participants were born outside the United States (32/123, 26%) and spoke non-English languages at home (55/123, 44.7%). Finally, more than half (65/123, 52.8%) of the participants had received therapy or counseling for a mental health condition in the past, but a majority (90/123, 73.2%) had not taken medication for a mental health condition in the past. Refer to Table 1 for more demographic information.

Using the symptom checklist cutoffs for the Patient Health Questionnaire-9, General Anxiety Disorder-7, and PTSD Checklist for the DSM-5, participants on average reported mild depression (mean 8.16, SD 5.74) and mild anxiety (mean 7.06, SD 5.60) and did not meet criteria for a probable PTSD diagnosis (mean 20.6, SD 18.0). The mean mental health literacy score was 117 (SD 9.14), indicating high mental health literacy. The mean score for internalized mental illness stigma was 13.2 (SD 7.33), suggesting low stigma. The mean score for attitudes toward mental health treatment was 57.4 (SD 6.48), indicating more positive attitudes toward mental health. The mean score for self-efficacy in seeking mental health care was 64.2 (SD 15.1), suggesting high self-efficacy.

**Table 1:** Sociodemographic characteristics of participants grouped by online tool use.

			Full sample (N=123), n (%)		Online tool use to seek mental health information				Online tool use to connect to professional service		
					Yes (n=61), n (%)		No (n=62), n (%)		Yes (n=37), n (%)		No (n=86), n (%)
**Gender**											
	Man	18 (15)		8 (44)		10 (56)		6 (33)		12 (67)	
	Woman	104 (85)		52 (50)		52 (50)		30 (29)		74 (71)	
	Agender	1 (0.8)		1 (100)		0 (0)		1 (100)		0 (0)	
**Race^a^**											
	Racial/ethnic minority	98 (80)		45 (46)		53 (54)		28 (29)		70 (71)	
	White	24 (20)		15 (63)		9 (37)		9 (37)		15 (63)	
**Ethnicity^b^**											
	Asian	47 (39)		20 (43)		27 (57)		12 (26)		35 (74)	
	Black or African American	5 (4)		1 (20)		4 (80)		1 (20)		4 (80)	
	Latinx	16 (13)		9 (56)		7 (44)		4 (25)		12 (75)	
	Middle Eastern	19 (16)		9 (47)		10 (53)		5 (26)		14 (74)	
	Multiracial	11 (9)		6 (55)		5 (45)		6 (55)		5 (45)	
**Non–US born status**											
	No	91 (74)		47 (52)		44 (48)		31 (34)		60 (66)	
	Yes	32 (26)		14 (44)		18 (56)		6 (19)		26 (81)	
**Language spoken at home**											
	English	68 (55)		35 (51)		33 (49)		23 (34)		45 (66)	
	Non-English	55 (45)		26 (47)		29 (53)		14 (25)		41 (75)	
**Previous therapy use**											
	Yes	65 (53)		38 (58)		27 (42)		27 (42)		38 (58)	
	No	58 (47)		23 (40)		10 (17)		10 (17)		48 (83)	
**Previous medication use**											
	Yes	33 (27)		24 (73)		16 (48)		16 (48)		17 (52)	
	No	90 (73)		37 (41)		21 (23)		21 (23)		69 (77)	

^a^Missing value for 1 participant (n=122).

^b^Breakdown of ethnicities for the category racial and ethnic minority (n=98).

### Quantitative Results

In total, 49.6% (61/123) of the participants used online tools to seek mental health information, while 30.1% (37/123) used online tools to find, be referred to, contact, or connect with a mental health professional or service.

#### Predictors of Online Tool Use to Seek Mental Health Information

The multiple logistic regression model was not statistically significant for online information seeking (N=86, *χ*^2^_12_=19.2; *P*=.08). Age, gender, race and ethnicity, non–US born status, past therapy use, symptom severity, stigma, self-efficacy, and attitudes toward mental health treatment did not predict online information seeking. However, mental health literacy was associated with an increase in the likelihood of online information seeking, such that odds of online tool use to seek mental health information increased by 14% for every additional increase in mental health literacy (odds ratio 1.14, 95% CI 1.05-1.24).

#### Predictors of Online Tool Use to Seek Mental Health Services

The multiple logistic regression model was not statistically significant for seeking mental health services online (N=86, *χ*^2^_12_=20.4; *P*=.06). None of the hypothesized predictors were associated with online help-seeking behaviors (Table 2).

**Table 2:** Regression analyses of predictors of online tool use.

			β (SE)		Wald *χ*^2^ (*df*)		*P* value		Odds ratio (e^β^; 95% CI)
**Predictors of online tool use to seek mental health information**									
	Age	−.07 (.07)		1.1 (12)		.30		0.93 (0.81-1.1)	
	Gender	.68 (.77)		0.8 (12)		.37		1.98 (0.44-8.9)	
	Race or ethnicity	−.22 (.68)		0.1 (12)		.74		0.80 (0.21-3.0)	
	Non–US born	−.10 (.62)		0.03 (12)		.87		0.90 (0.27-3.1)	
	Previous therapy	.59 (.64)		0.9 (12)		.35		1.8 (0.52-6.3)	
	Depression	.02 (.08)		0.04 (12)		.85		1.0 (0.87-1.2)	
	Anxiety	−.12 (.10)		1.6 (12)		.22		0.89 (0.73-1.1)	
	PTSD^a^	.03 (.03)		1.0 (12)		.34		1.0 (0.97-1.1)	
	Mental health literacy	.13 (.04)		12.4 (12)		.002		1.14 (1.1-1.2)	
	Internalized stigma	.01 (.05)		0.1 (12)		.74		1.02 (0.92-1.1)	
	Attitudes toward mental health treatment	−.03 (.06)		0.3 (12)		.59		0.97 (0.87-1.1)	
	Self-efficacy	−.01 (.02)		0.2 (12)		.69		0.99 (0.94-1.0)	
**Predictors of online tool use to connect with professional mental health services**									
	Age	.13 (.09)		2.7 (12)		.15		1.14 (0.95-1.4)	
	Gender	.43 (.84)		0.3 (12)		.61		1.54 (0.30-8.0)	
	Race or ethnicity	.78 (.79)		1.0 (12)		.33		2.18 (0.46-10.3)	
	Non–US born	.82 (.76)		1.2 (12)		.28		2.26 (0.51-10.1)	
	Previous therapy	−.31 (.74)		0.2 (12)		.67		0.73 (0.17-3.1)	
	Depression	−.19 (.10)		4.5 (12)		.05		0.83 (0.68-1.0)	
	Anxiety	.04 (.11)		0.1 (12)		.74		1.04 (0.84-1.3)	
	PTSD	.06 (.03)		3.9 (12)		.07		1.07 (0.99-1.2)	
	Mental health literacy	−.02 (.04)		0.3 (12)		.61		0.98 (0.91-1.1)	
	Internalized stigma	.04 (.06)		0.5 (12)		.47		1.04 (0.93-1.2)	
	Attitudes toward mental health treatment	.08 (.07)		1.6 (12)		.22		1.09 (0.95-1.2)	
	Self-efficacy	.01 (.03)		0.2 (12)		.63		1.01 (0.96-1.1)	

^a^PTSD: posttraumatic stress disorder.

### Qualitative Results

#### Types of Online Tools Used

Qualitative results indicated that participants used search engines, medical websites (eg, BetterHelp and Psychology Today), informational websites, apps (eg, MindDoc, LiveHealth, and Calm), and university-affiliated tools to seek mental health information and connect to services. Refer to Table 3 for a summary of qualitative responses.

**Table 3:** Summary of qualitative responses on types of online tools used for seeking mental health information and connecting with professional services (N=123).

Tool type codes	Definition	Seek mental health information^a^ (n =61), n (%)	Connect with professional services^b^ (n =37), n (%)
Search engines	Tools that carry out web searches to identify websites that correspond to given keywords	20 (48)	3 (8)
Medical websites	Websites with information related to mental health diagnosis, symptoms, or professional services	12 (20)	11 (30)
Informational websites	Websites that provide general background information and research on mental health disorders and coping mechanisms or exercises	6 (10)	2 (5)
Apps	Downloadable apps that can be used for mental health information, services, or resources	5 (8)	4 (11)
Discussion forums	Internet forums or message boards where an individual can post about issues or reply to other individual’s posts that they may find relatable	4 (7)	—^c^
Social media	Apps that can be used to share content	1 (2)	—
University-affiliated tools	Tools created by universities to provide mental health services or information to students	1 (2)	9 (24)
Call, video, and SMS text messaging	Platforms that can be used to connect with family, friends, or professionals through voice, video, or messages	—	4 (11)
Insurance	Tools created by insurance companies to be used for insurance-covered services and information	—	3 (8)
Vague statements	Statements that are too vague to code under a given category	3 (5)	1 (3)

^a^Codes endorsed for seeking mental health information online.

^b^Codes endorsed for connecting with professional mental health services online.

^c^Not applicable.

#### Reasons for Using Online Tools

Qualitative findings yielded 3 themes related to the reasons why participants used online tools. Participants used online tools because they (1) sought psychoeducation; (2) recognized a mental health need; and (3) perceived online tools as beneficial due to increased accessibility, usability, and availability of support.

A primary reason participants used online tools was to increase their knowledge and to access psychoeducation about mental health. Online tools, such as discussion forums and social media, were frequently used for seeking mental health information, including gaining a better understanding of their own mental illness (eg, understanding PTSD and anxiety symptoms) and due to having a general interest in mental health topics. For example, participants stated that they wanted to “better understand PTSD and ways to cope with anxiety” and to learn more about their “symptoms at the time and to see if they can explain any other bodily functions (ie, pain or aches, inconsistent menstruation cycles, and headaches).” Participants also used online tools to learn about coping skills that they could use to manage their mental health concerns. Participants reported wanting to learn about a variety of coping skills, such as exercise, sleep, nutrition (eg, “vitamin intake”), and “emotion regulation and meditation skills.”

Another reason participants used online tools to seek mental health information and connect with a mental health service was due to perceived need concerns, such as recognizing a mental health need and noticing that their symptoms were becoming more severe. For example, a participant reported, “I had been having anxiety that led to a panic attack and decided I needed to seek help in managing my anxiety,” while another mentioned that “I was having depersonalization panic attacks and did not understand what was happening to me.” Participants also used online tools, such as voice, video, and messaging tools, along with tools created by insurance companies, to connect with mental health services. Participants reported actively seeking help, expressing their intent to “find a therapist” and choosing online platforms because they “knew this is where” they “could find a mental health professional.” Participants stated that these online tools were often “recommended by someone trusted,” such as friends or family, who were aware of the participants’ mental health needs.

Participants also used online tools to find mental health information and connect with services because they found them beneficial. Many participants cited accessibility as a key factor, noting that they often could not find other traditional support and resources in a timely manner and instead turned to online tools because “it was fast, easy, and free.” Other participants highlighted usability as a key factor, describing online tools as convenient and intuitive, with a participant stating, “It’s convenient and I don’t need to ask someone else” and another expressing, “It’s quick and easy, and I can look for any information and get different input.” In addition, some participants used online tools to connect with others who shared similar mental health experiences, seeking a sense of community and emotional support “to feel less alone.” Refer to Table 4 for summary of themes and codes related to reasons for using online tools.

**Table 4:** Summary of qualitative themes and codes related to reasons for using online tools and perceived helpfulness (N=123).

Theme and code				Definition		Seek mental health information^a^ (n =61), n (%)		Connect with professional services^b^ (n =37), n (%)
**Reasons for using online tools**								
	**Sought psychoeducation**							
		Knowledge about personal symptoms	Wanting to learn more about mental health disorders and their symptoms		19 (31)		—^c^	
		Generalized interest	Had a generalized interest in learning more about mental health		10 (16)		—	
		Coping skills	Searching for techniques, strategies, or exercises to help cope with mental health issues or boost self-efficacy		9 (15)		—	
	**Perceived benefits: accessibility, usability, and availability of support**							
		Accessibility	Increased access to mental health services, information, or resources and usability		8 (13)		9 (24)	
		Lack of availability	Other resources being unavailable		1 (2)		1 (3)	
		Connecting with community	Wanting to connect with others with similar mental health issues, wanting to not feel alone, or searching for a sense of community		4 (7)		—	
	**Perceived need**							
		Symptom severity	Feeling a heightened personal struggle with mental health, elevated symptoms, or using tools as a last resort		6 (10)		6 (16)	
		Testimonial	Receiving a recommendation to use an online tool from another individual		1 (2)		2 (5)	
		Help-seeking behaviors	Vague statements wanting to generally seek help from professionals or services		1 (2)		17 (46)	
		Vague statements	Statements were too vague to code under a given category		2 (3)		2 (5)	
**Helpfulness of online tools**								
	**Provided valuable psychoeducation**							
		General information	Provided broad information about mental health		18 (31)		3 (9)	
		Knowledge from personal experience	Provided information about mental health disorders and their symptoms, so the individual was able to better understand		16 (27)		—	
		Coping mechanisms	Provided techniques, strategies, or exercises to help cope with mental health issues		11 (19)		—	
		Normalizing mental health	Normalized mental health disorders, symptoms, and treatments and boosted the individual’s confidence in their ability to improve mental health		3 (5)		—	
		Confirmed previous knowledge	Provided information that confirmed what they already knew about mental health issues		1 (2)		—	
		Help-seeking behaviors	Provided resources to seek help through a professional or service		1 (2)		15 (44)	
	**Attainable and convenient support**							
		Accessibility	Provided resources or services in an easily accessible manner		1 (2)		8 (24)	
		Specialty filter	Provided a tool to filter through website content to cater it to individuals’ needs		—		2 (6)	
		Delayed care	Eventually provided connection to care but had some challenges and barriers		—		3 (9)	
		Connections with community	Provided a connection to others with similar mental health issues or made the individual feel less alone		5 (9)		—	
		Vague statements	Statements that are too vague to code under a given category		3 (5)		3 (9)	

^a^Codes endorsed for seeking mental health information online.

^b^Codes endorsed for connecting with professional mental health services online.

^c^Not applicable.

#### Helpfulness of Online Tools

Most participants reported that the online tools they used to seek mental health information were somewhat (50/61, 82%) and very (9/61, 15%) helpful. Online tools used to connect to services were also reported to be somewhat (16/37, 43%) and very (18/37, 49%) helpful.

Qualitative findings yielded 2 themes related to the reasons why participants found online tools helpful. Online tools were perceived as helpful because they (1) provided valuable psychoeducation and (2) made obtaining support more attainable and convenient.

Students found online tools to be helpful because they provided information about symptoms of mental health disorders (eg, anxiety, depression, and panic attacks), coping mechanisms (eg, techniques to calm down), general mental health facts (eg, “Provided me with more information than I originally had”), and ways to seek help (eg, resources on using university counseling and psychological services). For example, a participant reported that online tools “allowed me to have more understanding of myself before seeking treatment” and “they helped me realize that these feelings were symptoms of anxiety and depression.” Given that psychoeducation was the primary reason for both using online tools and finding them helpful, this indicates that most participants who used online tools were able to find the information they were initially searching for. Some participants expressed that online tools were also helpful because the psychoeducation normalized mental health (eg, “They helped normalize my behavior and gave me the confidence to make progress with my CBT”) and confirmed previous knowledge they had about mental health (eg, “Didn’t tell me new information, just confirmed what I already had expected”).

Participants expressed that online tools were helpful because they made obtaining support more attainable and convenient, with a participant mentioning, “It was a lot easier to schedule a time,” and other participants stating, “it gave me support when I needed it.” Participants also expressed that online tools were easy to use, with a participant explaining, “The site was extraordinarily simple to use.” Certain features, such as specialty filters, made support more attainable, with a participant stating, “I was able to specify my search via provider specialty and modality.” However, a couple of participants experienced delays in accessing care, as a participant mentioned, “It took so long to go through the list, but I eventually found someone.” In addition, online tools made it easier to connect to others who were dealing with similar feelings or symptoms, resulting in participants feeling less alone. For example, a participant shared, “It is nice to know that others feel the same way that you do and that you can feel more normal because of it.” On the basis of these responses, participants who had aimed to connect to community and mental health services using the online tools found that they were able to effectively do so. Refer Table 4 for themes and codes related to online tool helpfulness.

#### Reasons for Not Using Online Tools

In total, 50.4% (62/123) of the participants reported that they did not use online tools to seek mental health information, and 69.9% (86/123) of the participants reported that they did not use online tools to connect with professional mental health services.

Qualitative findings yielded 3 themes related to why students did not use online tools. Participants did not use online tools due to (1) a lack of awareness about existing online tools, (2) negative attitudes toward online tools, and (3) a lack of perceived need.

Many participants refrained from using online tools to seek mental health information or connect with services because they did not know they existed. Participants reported a lack of awareness about suitable platforms, with some explaining they “have not seen them” or “didn’t know of any” online tools for mental health information or connecting to services.

For participants who were aware of online tools, they expressed not using online tools because they did not “trust them” and because they believed that online tools were “not personalized or accurate.” Along with finding online tools unreliable or unhelpful, some participants disclosed a preference for traditional help, such as already having a “personal therapist” or receiving in-person referrals from trusted individuals (eg, primary care providers, family, and friends).

Another commonly reported reason for not using online tools to find mental health information or connect to services was due to a lack of perceived need, as participants reported that mental health help was unnecessary or that they were not dealing with severe symptoms. For example, participants stated, “I don’t believe that I have needed to connect with a professional/service” and “I don’t think it has reached a point for me where I need to seek professional help.” Others expressed avoidance and self-reliance tendencies, explaining that they did not “put much thought into learning about mental health” or “how to feel better,” and that “I tend to deal with any problems I have by myself.” Refer to Table 5 for a summary of themes and codes explaining why online tools were not used.

**Table 5:** Summary of themes and codes explaining why online tools were not used to seek mental health information and connect with professional services, as well as factors motivating increased use (N=123).

Theme and code				Definition		Seek mental health information (n =62), n (%)		Connect with professional services (n = 86), n (%)
**Reasons for the lack of use**								
	**Lack of awareness**							
	Lack of awareness		Reasoning related to not knowing tools existed or which tools to use		12 (19)		10 (12)	
	**Attitudes toward online tools**							
		Unreliable	Reasoning related to not finding tools to be reliable or trustworthy		8 (13)		2 (2)	
		Inaccessible and unhelpful	Reasoning related to ease of tool use or believing the tools would not be helpful		5 (8)		4 (5)	
		Traditional help preference	Reasoning related to preferring to talk to a professional or trusting a professional more		10 (16)		19 (23)	
	**Lack of perceived need**							
		Mental health help is unnecessary	Reasoning related to broadly believing mental health help or services to be unneeded, so no online tool was used		11 (18)		28 (34)	
		Low symptom severity	Reasoning related to not experiencing mental illness or any problems with mental health		10 (16)		10 (12)	
		Avoidance	Reasoning related to not wanting to resurface negative feelings or deal with problems		2 (3)		—^a^	
		Self-reliance	Reasoning related to dealing with mental issues by themselves or on their own		1 (2)		1 (1)	
		Vague statements	Statements that are too vague to code under a given category		3 (5)		9 (11)	
**Motivating factors**								
	**Reliability**							
		Recommendation	Suggestions related to referral from friends, family, or professionals; support and encouragement; and positive reviews and testimonials		19 (31)		19 (22)	
		Trustworthiness	Suggestions related to reliability and confidentiality		4 (7)		4 (5)	
	**Accessibility, availability, and usability of support**							
		Specific features	Suggestions related to specific website and app features, technological services, personalization, and usability		10 (16)		7 (8)	
		Accessibility	Suggestions related to finances, time, and other aspects of increased accessibility		6 (10)		10 (12)	
		Awareness	Suggestions related to advertising or promoting awareness		4 (7)		8 (9)	
	**Perceived need**							
		Symptom severity	Suggestions related to an increase in negative symptoms or behaviors or an increase in negative impact on everyday life		6 (10)		13 (15)	
		Help seeking	Suggestions related to broadly wanting to seek help		2 (3)		3 (4)	
	**Personal preferences**							
		No motivators	Includes reasoning related to individuals currently in therapy or services		4 (7)		8 (9)	
		Vague statements	Statements that are too vague to be coded under a given category		6 (10)		14 (16)	

^a^Not applicable.

#### Motivators for Using Online Tools in the Future

Of participants who reported not using online tools, a majority expressed motivation to use online tools to seek mental health information (58/62, 94%) or connect with services (78/86, 91%).

Among those who did not use online tools, 3 key factors emerged as potential motivators for future use. Participants expressed they would be motivated to use online tools for their mental health in the future if (1) they perceived a mental health need, (2) they trusted that the tools were reliable, and (3) the tools were accessible and easy to use.

Participants expressed that they would be motivated to use online tools if they found their symptoms to be severe or if their symptoms started to impact their functioning. For example, students stated that they would use online tools “If stress/depressed state impacted my other responsibilities and friends/family” and “if my anxiety keeps getting worse after the school year ends.”

Participants expressed that recommendations from trusted individuals, such as “a professional,” “family or friends,” or a “therapist,” would motivate them to use online tools, as recommendations would indicate that the online tool was reliable and trustworthy. One participant stated they would use an online tool “if I heard a good review from people I know,” while another added, “if a professional gave me some to use.” Other participants noted encouragement from others would also increase the likelihood of using online tools to seek help, such as “knowing that it’s encouraged to reach out for help may make me feel more inclined to reach out if I found myself in the position.”

Regarding accessibility, participants reported that they would be open to using online tools if they were “free” or “not expensive” and if the online tools used “easy to understand language” and provided “easily accessible information.” Many participants also emphasized that raising awareness of the online tools would improve their accessibility, stating “awareness of their existence and that they have proven to be effective” and “more advertisement” as key motivators. Finally, students suggested that specific features improving the usability of the online tool, such as “video chats, scheduled appointments, surveys,” or “a comprehensive search filter on the app so you can find the best therapist for your needs” would make them more willing to use the online tool in the future. They also emphasized that the online tool should have “no association to emails, names, or locations” to improve usability. Refer to Table 5 for additional motivating factors that would encourage the use of online tools for mental health.

## Discussion

### Principal Findings and Comparison to Previous Work

Due to the high prevalence of mental health problems, low use of mental health services among university students, and the rise of the internet, there has been an increase in online tools available for mental health, including online tools to reach and connect youth to mental health services [2,5]. However, the availability of digital tools for mental health has not translated to widespread use [44]. Our study found that about half of the participants (61/123, 49.6%) used online tools to seek mental health information, and only a third (37/123, 30.1%) used online tools to connect with mental health professionals or services. Regarding the types of online tools used, our findings are consistent with previous reports stating that a high proportion of young people use search engines to locate mental health resources, with smaller percentages using platforms such as discussion forums and mental health apps [8]. Our study also found much lower rates of using these online tools to actively seek professional mental health help, in comparison to mental health information [8].

We found that students who had higher mental health literacy were more likely to use online tools to seek information but not professional help. This might suggest that students with more knowledge on mental illness, such as the ability to recognize and label their symptoms or attribute them to mental health rather than other causes, may be better equipped to seek help online. For example, students with higher mental health literacy may know what specific key terms to use to search for relevant information. In addition, those knowledgeable about coping skills (eg, relaxation skills for anxiety) may be more likely to use online tools to find more information on how to practice these skills. Although higher mental health literacy is associated with increased intentions to seek help, it might not translate to actual service use [45]. Considering the cross-sectional nature of our study, it is also possible that greater use of online tools may improve the mental health literacy of students by increasing exposure to online information. The lack of association between mental health literacy and the use of online tools to connect with professional services may be due to individuals interpreting online psychoeducational resources and coping strategies as sufficient, leading them to conclude that their symptoms did not require professional care [44,46]. This may also reflect students’ perception of digital mental health tools as either a more accessible alternative to traditional services or as a complement to conventional care [7].

Our study found that mental health symptoms, including depression, anxiety, and trauma symptoms, did not predict online tool use. Past research has found mixed findings on this association, with 1 study finding anxiety symptoms to predict app use, while depression did not [12]. It is possible that we did not find this association because participants were asked about overall online tool use and not specific online tools, such as apps. Moreover, students may be using different types of online tools available (eg, websites, apps, and smart devices) depending on different symptom-related concerns. For example, some individuals may prefer using websites rather than an app for information seeking and psychoeducation on symptoms, while others may prefer self-help apps that provide tips to regulate symptoms and promote relaxation [12]. Although mental health symptoms were not statistically significant predictors of online tool use, given that some associations trended toward significance (depression: *P*=.05, PTSD: *P*=.07), it may be possible that the study was too underpowered to detect a statistically significant relationship between mental health symptoms and online tool use.

Furthermore, our sample reported mild depression and anxiety on average, with very few PTSD symptoms. On the basis of the qualitative findings, a common reason for not using online tools was a lack of perceived need. Therefore, it is possible that the absence of an association between mental health symptoms and online tool reflects students’ belief that their symptoms did not warrant significant attention. Other evidence indicates that young people with more severe symptoms, especially those with higher depression severity, may avoid traditional care, preferring to use anonymous electronic sources (ie, internet forums, support groups, and chat rooms) and digital tools to better understand and cope with their symptoms on their own [47]. Considering students who have more severe symptoms of depression may not seek care compared to those with more moderate symptoms due to coping with specific depression symptomology (ie, lack of motivation, low energy, increased fatigue, and reduced problem-solving abilities), such self-reliance may further reduce their perceived need for professional mental health services [48,49]. Future research should examine what factors may play a role in students’ perceived need of mental health care.

Participants in our study reported that online tools to seek information were accessible, easily providing fast information and answers to questions quickly, which is consistent with past research [21,50,51]. While previous research had mixed results about existing disparities in online tool use based on race or ethnicity, non–US born status, and gender, our study found no differences in online tool use based on these factors [7,16,23]. However, it is important to note that our sample was highly educated, had lower stigma, and had more positive views and knowledge about mental health than the general population. These factors likely facilitated the use of online tools for mental health among our participants. Although we did not find associations between stigma, attitudes toward mental health treatment, or self-efficacy and online tool use among our sample, this may be a result of the privacy provided by the digital nature of these tools. While support from family and friends may be incorporated in digital tools to promote the management and treatment of mental health disorders, for those dealing with more social or family-based stigma, the anonymity and privacy of online tools may provide more autonomy to decide who they would like to involve in this process, if any [7,52,53]. For those who do not feel comfortable using online tools, it may be due to harboring certain attitudes and beliefs about digital tools, such as finding them untrustworthy or unreliable, that resulted in their unwillingness to use them. As for self-efficacy, digital self-efficacy, such as students’ confidence in navigating digital spaces, may have a greater impact on their willingness to use online tools rather than their confidence in seeking mental health support itself [18]. Therefore, stigma that exists around the confidentiality of online data, negative attitudes toward the use of digital platforms, and students’ confidence in their ability to navigate them effectively may play a role in online help seeking. This highlights the importance of research examining the different types of attitudes and beliefs that exist about digital mental health tools, specifically.

### Recommendations to Improve the Implementation of Online Tools

Due to possible differences in barriers faced by students across a variety of school settings, we recommend that researchers consider the needs and priorities of their target population to increase the relevance and fit of the digital tools. It is important to recognize that students from other educational settings, aside from 4-year institutions, may experience barriers to using online tools for mental health. For example, research has found that community college students face many barriers to using digital tools (eg, mental health apps), including financial challenges, privacy concerns, self-reliance, skepticism about the seriousness of symptoms, and concerns about others’ perceptions of them [51]. This suggests that accessibility and stigma barriers may exert a more pronounced impact on community college students than on their university counterparts [51]. Thus, it is important to consider the target population and setting when implementing digital tools. To develop digital tools that are acceptable to college students from a variety of educational backgrounds, we recommend that formative research be conducted with the population of interest, such as identifying primary mental health concerns and including user content and interface suggestions (ie, crisis text line, telehealth, websites for connecting to services, SMS text messaging with counselors, personalization, anonymity, privacy, security, and peer engagement) that may promote service use before the implementation and dissemination of a digital tool [8,54,55].

Our qualitative results demonstrated that many students are unaware of how digital tools could be used to connect with care. There is a need to improve the digital tool’s user interface because many online tools do not have the ability to connect users directly to a coach or therapist [44,56]. Given that students view the ability to connect to care as important, we recommend that online tools help users assess symptoms and include a direct connection to professional services, as it may increase use of mental health services among college students with unmet mental health needs [12]. For example, rather than having students navigate help seeking on large search engines, which may be a dissatisfying process, digital tools that could triage help should be promoted and researched, as highlighted by our participants’ requests for features that personalize the help-seeking process. Young people have reported higher satisfaction when online tools such as Link, a navigation website that connects youth to treatment based on reported mental health symptom type and severity, were tailored specifically to their needs (ie, immediate return of personalized options for help seeking, stories from individuals dealing with similar symptoms, and self-care tips) [57]. Digital interventions, such as the Screening and Treatment for Anxiety and Depression program that screens college students for mental health symptoms and reliably connects students to evidence-based care (eg, self-guided wellness program, sessions with peer coaches, or individual therapy) based on the severity of depressive symptoms, might act as a gateway to connect students to care [49]. This type of online tool could circumvent barriers such as misjudging perceived need and self-reliance, facilitating a more efficient pathway for students to access the necessary mental health support [49].

Qualitative findings also indicated that students used online tools for psychoeducation and general information about mental health and found them helpful. Although there are websites that provide general psychoeducation, such online tools may lack detailed information about evidence-based treatments and guidance on connecting with professionals [8,12,58]. Without adequate psychoeducation on available treatments and how to access them, students may struggle to take the next step in pursuing professional services, ultimately leaving their mental health needs unmet. College students have reported that online platforms, such as clinic websites, should provide more comprehensive information, including clear explanations on how to get started with services, detailed descriptions of services available for specific mental health disorders, and mental health professionals’ background information, which in turn can increase a students’ willingness to seek mental health care [59]. Thus, we recommend that digital tools provide explicit psychoeducation on how to receive evidence-based treatments, as that may increase students’ likelihood of using online tools to connect to mental health services. Another recommendation for content creators who design mental health websites is to include resources for crisis lines, which was suggested by participants who did not use online tools to connect to care as a possible motivating factor for future use. Crisis lines have been shown to connect people with immediate help and promote engagement with long-term mental health services (ie, referrals to mental health professionals) [60,61].

### Recommendations to Increase the Dissemination of Online Tools

Our qualitative results demonstrated that a major reason why college students did not use online tools to support their mental health was because they were unaware of what digital tools were available. Indeed, previous studies have found that many participants are unaware of formal online resources (ie, charities and health services) available to support them [8]. Considering we found mental health literacy to predict online tool use, video interventions and digital media campaigns targeting young people with low mental health literacy should also include information on available online tools for mental health support. These interventions can enhance mental health literacy, reduce stigma, and promote help seeking by increasing awareness of various online tools for accessing care [62].

Referrals from friends, family, or health care professionals could help raise awareness about available online tools. As support from loved ones often encourages help seeking, recommendations from these sources signal to participants that the tools are more reliable and trustworthy [7,44,55]. Although numerous digital tools have become more readily available, proper evaluation and validation of these tools and their implementation is limited, resulting in students needing to deduce whether a tool is trustworthy or not [63]. While young people may be motivated to search the internet for mental health help, their ability to deduce the quality and reliability of the mental health information may prove to be more challenging with limited mental health literacy, further hindering their willingness to use online tools. It is recommended that mental health professionals receive training on available digital tools so they can confidently recommend them to patients, who may then share these tools with friends and family [43,63,64]. Therefore, referrals and recommendations by trusted individuals can spread awareness of credible digital tools, and in turn, reach underserved populations who may have negative perceptions of the internet and digital mediums or find the digital tools unreliable due to privacy concerns [13,14,45,55,65].

### Limitations and Future Directions

As data for this study were collected in 2021, findings should be interpreted within the unique context of the COVID-19 pandemic. Although our study found that a third of participants (37/123, 30.1%) used online tools to connect with mental health professionals or services, which was consistent with online help-seeking rates before the COVID-19 pandemic [21], general use of online tools to seek mental health information likely increased during the pandemic [8,25,27]. At that time, lockdowns and social distancing measures significantly limited in-person mental health services, which may have influenced students’ decisions to seek information and support through online tools. These conditions may have contributed to both increased exposure to and reliance on online mental health resources. Consequently, patterns of online help seeking captured in this study may reflect a period of heightened necessity rather than sustained preference. Future research is needed to examine how students’ attitudes and behaviors toward online mental health tools have evolved after the pandemic, when the use of digital resources is increasingly optional rather than mandated.

We used standardized procedures to code the qualitative responses; however, some open-ended responses were too vague to be coded (eg, “it was something” was provided as a response to “Why was this online tool helpful?”). In addition, our small sample size limited our ability to quantitatively examine patterns in types of online tools used, perceived helpfulness, and motivating factors across different demographic groups. To better tailor these tools to populations considered marginalized, we believe that future research should aim to identify if there are differences in these outcomes based on demographic characteristics. Related to our sample size, post hoc power analyses suggest the study may have been underpowered to detect small to moderate associations between various key predictors and outcomes. For example, analyses examining the relationship between mental health symptoms (depression, anxiety, and trauma symptoms) and online tool use were underpowered (eg, depression: power=0.69, anxiety: power=0.10, and trauma: power=0.63), potentially limiting our ability to detect significant effects. Our study sample also had a positively skewed distribution of mental health symptoms, with most participants reporting mild symptoms, as well as a negatively skewed distribution for self-efficacy and attitudes, where most participants expressed high self-efficacy and more positive attitudes toward mental health. Future research with larger, more diverse samples and greater variability in responses is needed to clarify whether these null findings reflect a true absence of association or limitations in statistical power. Due to the cross-sectional design of our study, the directionality of associations between our key predictor variables and online tool use also could not be established. Future longitudinal or experimental research is needed to clarify causal pathways and better understand the temporal sequencing of these relationships.

Furthermore, this study did not measure the following factors that are associated with the use of online tools for mental health: access to reliable technology, digital literacy (ie, ability to locate, evaluate, and communicate information using digital mediums), internet self-efficacy (ie, confidence in one’s ability to master new technology), attitudes toward digital mental health (ie, positive and negative views relating to trustworthiness, credibility, and efficacy), and preferences for in-person help seeking [11,12,15,65,66]. Future research should examine how these factors play a role in the use of online tools for mental health purposes, as highlighted by our qualitative responses. In addition, participants in our sample were recruited from students enrolled in psychology courses and may be more knowledgeable about mental health and have less stigma than students with other majors [67,68]. These findings are limited to the study sample, which included university students attending a 4-year institution, and may not be applicable to students from other educational backgrounds.

### Conclusions

There is a need to bridge the gap between effective online tools for mental health and users. Research exploring digital mental health tool design, acceptability, and feasibility can promote access to psychological information and professional services among college students [10]. Our study shows that university students find online tools to be an acceptable and feasible method to seek mental health information; however, more research is needed on how to optimize online tools to help students connect to mental health care. To optimize the utility of online tools, it is crucial to conduct research that will yield rich user insight to identify and implement user-suggested components, such as psychoeducation about disorders, treatments, and services; personalized content; interactive features; and connections to professional care [9,69-71].

## Abbreviations

PTSD: posttraumatic stress disorder
UCLA: University of California, Los Angeles
